# 

**Published:** 1868-07

**Authors:** 


### MEDICAL EXCHANGES (IN PART.)

BOSTON MEDICAL AND SURGICAL JOURNAL, $4 a year, published by David Clapp & Son, 334 Washington street, Boston, Mass. Now in its 75th volume.

BUFFALO MEDICAL AND SURGICAL JOURNAL, $3 a year, edited by Julius F. Miner, M. D., Surgeon to the Buffalo General Hospital. Address Medical and Surgical Journal, Buffalo, N. Y.

CHICAGO, (ILL.,) MEDICAL EXAMINER, $3. N. S. Davis, M. D. Ed.

DENTAL COSMOS, a monthly record of dental science, devoted to the interests of the profession, edited by Drs. McQuellan & Zilgler, $2.50 a year, single Nos. 25c. Philadelphia, Pa., 528 Arch street; 658 Broadway, New York; 16 Tremont Row, Boston; 100 Randolph Street, Chicago, Ill.

DENTAL REGISTER, Cincinnati, Ohio, published monthly for $3.00 a year, 167 Walnut St.

HOMOEOPATHIST AMERICAN, Published monthly by Smith and Worthington, 21 west 4th St, Cincinnati, Ohio. E. B. Thomas, M. D. Editor, $1.50 a year.

HERALD OF HEALTH, and Journal of Physical Culture, $5 a year, single Nos. 20 c., published by Miller, Wood & Co., 13 and 15 Laight street, N. Y. W. Tweedie, 337 Strand, London.

LONDON LANCET, Republished simultaneously by Wm. C. Herald, No. 32 Beekman st. New York, $5 a year, postage 24 cts. a year. Supplied to the trade by the American News Co. 119 and 121 Nassau St. New York.

MEDICAL JOURNAL, Chicago, Ill., $3. J. A. Adams, M. D. L. L. D. Editor.

MEDICAL REPORTER, a semi-monthly record of medicine and surgery, edited by Drs. J. S. B. Alleyne and O. F. Potter, $3 a year; St. Louis, Mo.

MEDICAL INVESTIGATOR, $2.00, Chicago.

PHRENOLOGICAL JOURNAL, $3 a year, 389 Broadway, New York. Sample Nos. 30 cts.

MEDICAL AND SURGICAL PIONEER, Kansas City, Mo., being a monthly record of medicine and surgery; $4 a year.

NASHVILLE, (TENN.,) JOURNAL OF MEDICINE AND SURGERY, by W. K. Bowling, M. D. $3 a year.

PACIFIC MEDICAL AND SURGICAL JOURNAL, $5. San Francisco, Cal., Bancroft & Co., Publishers. Henry Gibbons, M. D. Editor.

